# 

**Published:** 1954-07

**Authors:** 


					VV I
!\C3S1 ' t
THE
medical journal
OF THE
SOUTH-WEST
Incorporating
The Bristol Medico-Chirurgical Journal
July 1954
VoL
' 70 (li!)- No. 2^5 Price 4/-

				

